# A case report of left atrial myxoma presenting as embolic stroke

**DOI:** 10.1002/ccr3.8022

**Published:** 2023-10-11

**Authors:** Prachi Sharma, Yusuf A. Kumble, Abhigan Babu Shrestha, Vikash Jaiswal

**Affiliations:** ^1^ LPS Institute of Cardiology Kanpur Uttar Pradesh India; ^2^ Indiana hospital and heart institute Mangalore Karnataka India; ^3^ M Abdur Rahom Medical College Dinajpur Bangladesh; ^4^ Department of Research and Academic Affairs Larkin Community Hospital South Miami Florida USA

**Keywords:** 2D echocardiogram, cardiac tumor, embolic stroke, left atrial myxoma

## Abstract

**Key Clinical Message:**

Embolic stroke may rarely be the first presenting symptom of atrial myxoma. Multiple infarcts should be evaluated for embolic causes. Correct etiological diagnosis in cardio‐embolic stroke guides proper management strategy. It reinforces on the importance of early echocardiogram in the initial evaluation of patients presenting with ischemic stroke.

**Abstract:**

Atrial myxoma is a benign cardiac tumor found most commonly in the left atrium in 80% of the cases. Almost 1 in 10 myxomas are familial and are most common in women. Cardiac myxoma mostly present with dyspnea, fatigue, or palpitations. Previously undiagnosed left atrial myxoma (LA) presenting as stroke is extremely rare. Authors describe the case of a middle‐aged man with LA myxoma presenting with acute ischemic embolic stroke that was surgically excised. This case report emphasizes on the rare presentation as embolic stroke and the role of cardiac imaging in patients presenting with ischemic stroke. Early and coordinated teamwork among the neurologist, cardiologist, and cardiothoracic surgeon help establish the etiology and provide appropriate treatment.

## Graphical abstract

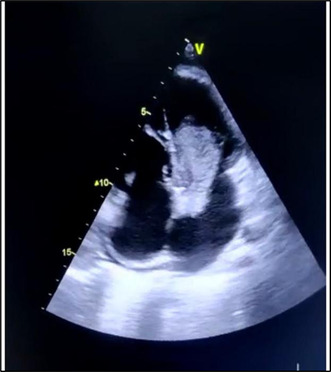

## 1 INTRODUCTION

Primary cardiac tumors are rare with a reported incidence of 1380/100 million individuals.^1^ Among primary cardiac tumors, approximately 75% are benign.^2^ Of the benign tumors, nearly half are cardiac myxomas, which predominantly arise from or near the interatrial septum and extend into the left atrium.^3^ It primarily affects the female population with a female‐to‐male ratio of 2:1.^4^ The etiology is unknown, and the only evidence pertaining is its mesenchymal origin.

It is often difficult to diagnose myxoma of the heart due to less symptoms or masked symptoms, and hence it should be considered as a differential diagnosis for unexplained syncope and dyspnea.^3^ The progression of the tumor can rarely lead to embolic stroke.^5^ The current guidelines of stroke management do not elaborate on cardio‐embolic stroke with myxoma, and only a few cases have been reported till date.^6^

## 2 PRESENTATION OF CASE

**FIGURE 1:**
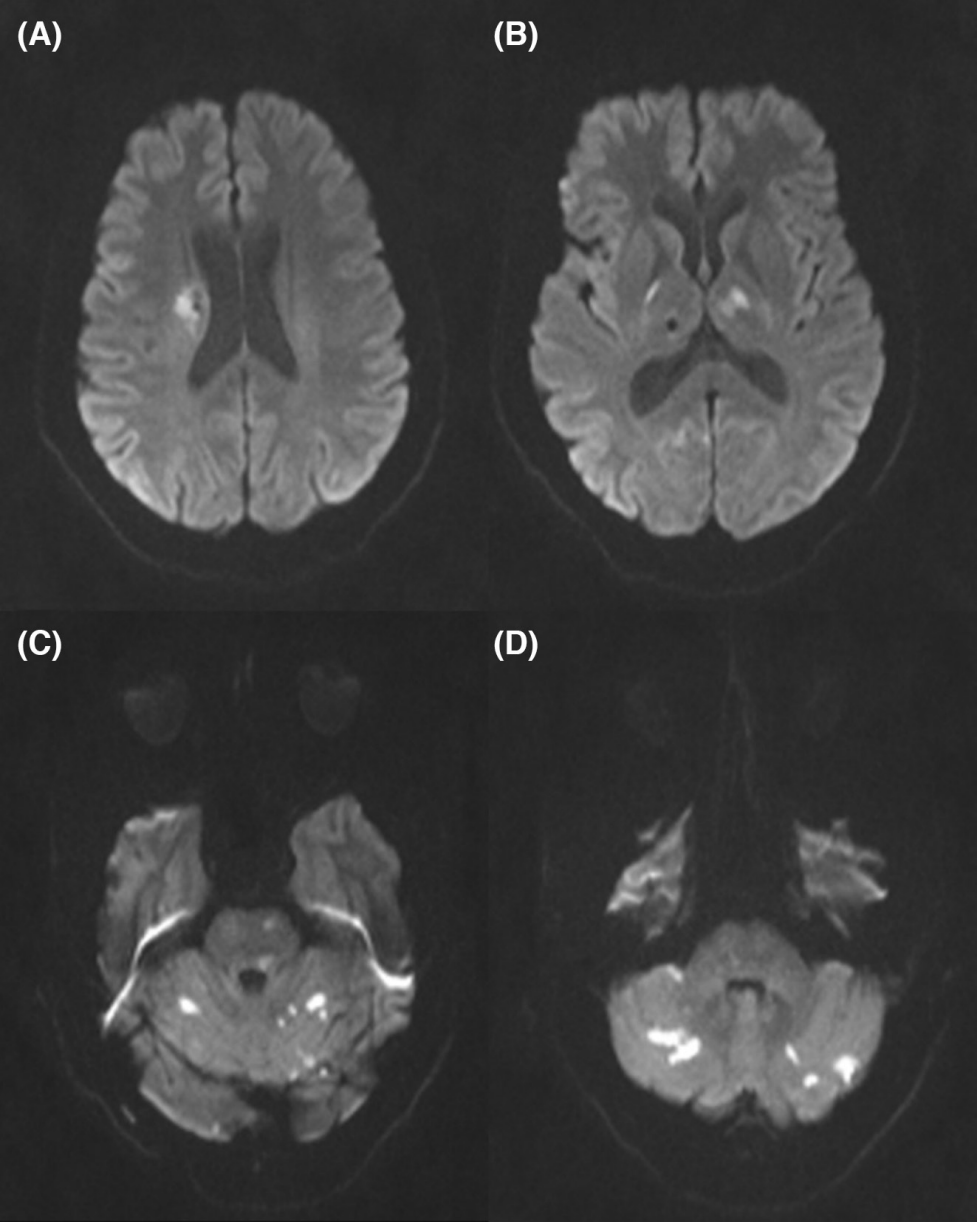
Multiple foci of high signal on diffusion weighted imaging seen in (A) right corona radiata, (B) left thalamus, and (C and D) bilateral cerebellar hemisphere.

We describe the case of a 48‐year‐old South Asian gentleman presenting to the emergency department with complaints of sudden onset of slurring of speech, episodes of vomiting, and complete motor aphasia for last 10 h which started improving slowly over the last 1 h. The patient had similar complaints 5 months back which resolved completely in a few hours, so the patient did not seek any medical advice. He had no history of hypertension or diabetes mellitus. On physical examination, the patient was afebrile. His blood pressure was 106/74 mmHg with a heart rate of 104 beats/minute and all distal pulses were felt. On cardiac examination, heart rhythm was regular, and a faint, short mid‐diastolic murmur was heard. Neurologically, there was motor aphasia with no peripheral neurological deficit. Computed tomography (CT) scan of the brain showed multiple infarcts in left parietal lobe, right occipital lobe, right body of caudate nucleus, left internal capsule, left thalamus, and bilateral cerebellar hemispheres. Magnetic resonance imaging (MRI) of the brain depicted multiple foci of high signal on diffusion weighted imaging (DWI) in right corona radiata, left thalamus, and bilateral cerebellar hemispheres making a diagnosis of infarcts (Figure 1). These multiple bilateral infarcts were suggestive of cardio‐embolic origin. However, the patient had no past relevant cardiac history.

**FIGURE 2:**
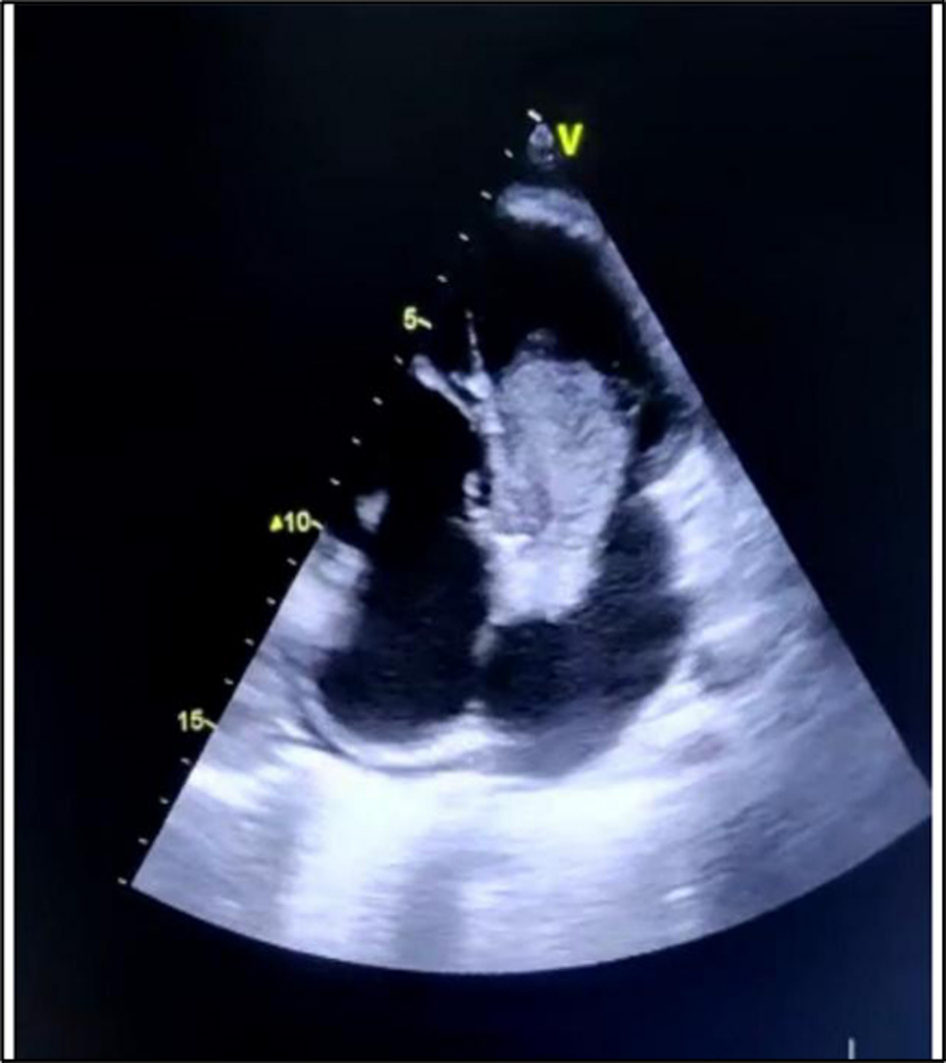
2D echocardiogram apical 4‐chamber view showing 39 × 20 mm left atrial myxoma protruding into the left ventricle across the mitral valve.

**FIGURE 3:**
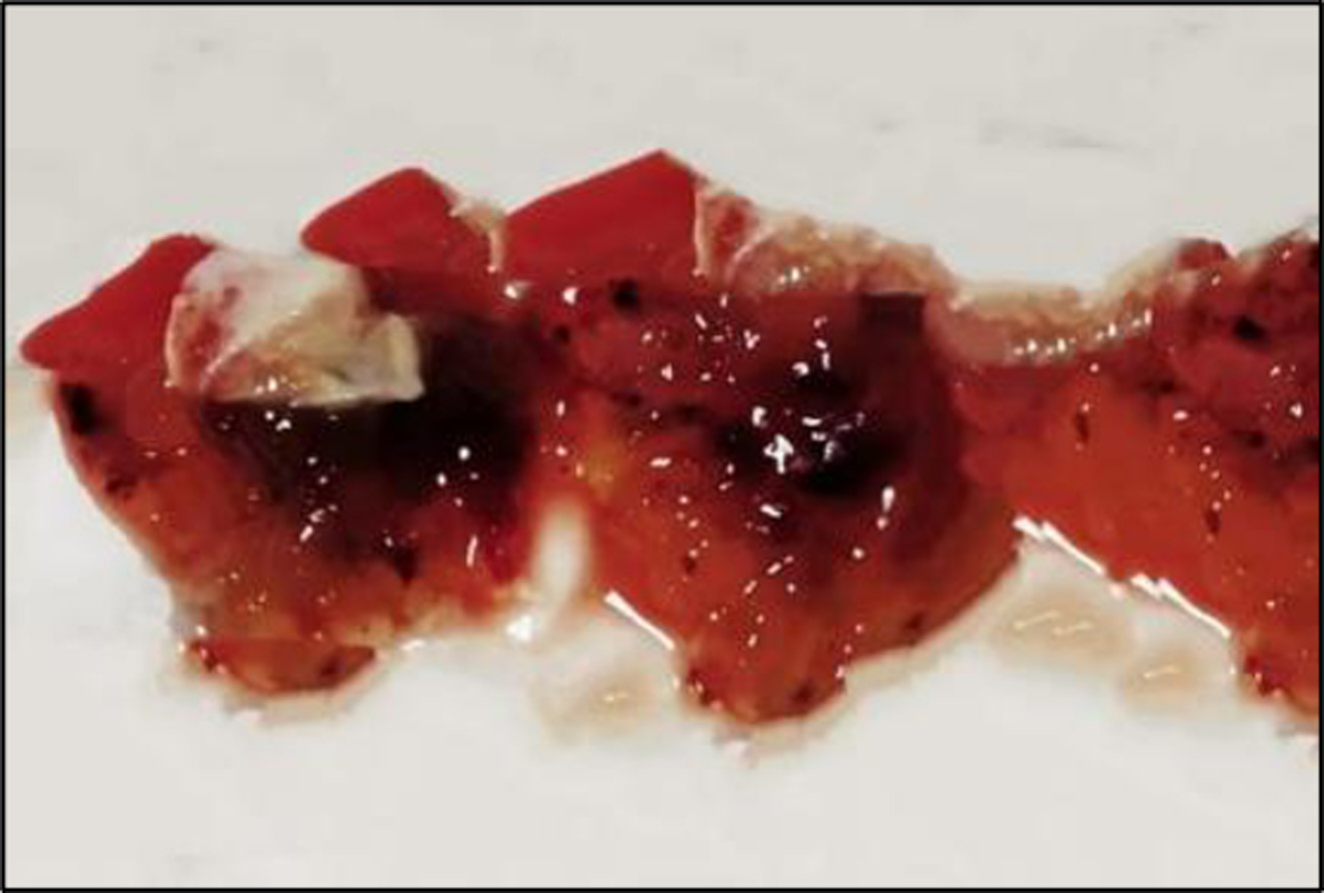
The excised myxoma.

Cardiac workup was done immediately. Electrocardiogram of the heart showed normal sinus rhythm with left atrial enlargement. Two‐dimensional echocardiogram was performed which revealed a 39 × 20 mm large homogeneous mass in the left atrium attached to the interatrial septum which was mobile and protruding into the left ventricle (Figure 2). The appearance was suggestive of a left atrial myxoma. In view of cardio‐embolic stroke, he underwent surgical excision of the mass (Figure 3). Biopsy report confirmed it to be a myxoma. He consequently recovered his normal speech and was discharged in a stable condition. On follow‐up, 4 weeks later, the patient was asymptomatic. 2D echocardiogram revealed no residual mass and normal cardiac chambers.

## 3 DISCUSSION

Atrial myxoma usually present with dyspnea, palpitations, or fatigue and is more common in women. Atrial myxoma presenting as stroke is a rare condition. However, it can potentially lead to varied neurological complications in 20%–35% of the patients.^5^ This case report emphasizes on the rare neurological manifestation of left atrial myxoma first presenting as an embolic stroke in a middle‐aged male patient, with no other cardiovascular symptoms. Usually, it obstructs the mitral inflow and causes fatigue and dyspnea due to reduced forward flow and elevated pulmonary venous pressure, respectively. There may be enlargement of the left atrium, atrial arrhythmias, and palpitations. Being a highly mobile and friable structure, its fragments may get dislodged and embolize to the cerebral circulation causing stroke. Bilateral multiple infarcts, as found in our case, usually point toward cardio‐embolic etiology. Although cardio‐embolic stroke is most commonly caused by left atrial thrombus due to valvular or non‐valvular atrial fibrillation. While, globally non‐valvular atrial fibrillation is the most common cause of cardio‐embolic stroke, in developing countries, rheumatic heart diseases (RHD) with severe mitral stenosis still remain a very common cause. Atrial myxoma is quite a rare cause for stroke, especially as the presenting symptom, like in our case.^7^

This case report serves as a reminder that can contribute to raise awareness and emphasize on the importance of quickly ruling out cardio‐embolic causes of ischemic stroke that are unrelated to hypercoagulability.^5^ In the absence of cardiac imaging, considering in situ thrombosis as the differential diagnosis, the patient could have been devoid of definitive treatment. It reinforces the need for a two‐dimensional echocardiography as a part of basic cardiac workup in patients presenting with stroke. In our patient, two‐dimensional echocardiography was performed which showed a 39 × 20 mm large homogeneous mobile left atrial mass attached to the interatrial septum. Multiple imaging characteristics—large size, attachment to the interatrial septum, mobility, and prolapse across atrioventricular valve—made myxoma the most likely diagnosis.

The diagnosis of an atrial myxoma warrants its resection, and it should be done immediately due to anticipated risk of embolization, arrhythmias, and sudden cardiac mortality occurring in about 10% of patients waiting for surgery.^8^ When it is resected completely with no residual tumor, the chances of recurrence are rare with good long‐term outcomes.^9^ Our patient had already suffered embolism, and in order to prevent further episodes, he was immediately taken up for surgical excision of the mass. Apart from imaging, histopathological findings are essential to confirm the diagnosis and provide prognostic information.^9^

## 4 CONCLUSION

Cardiac imaging in the form of 2D echocardiogram is an essential tool in the evaluation of ischemic stroke. Rarely, left atrial myxoma may present as embolic stroke. Embolism warrants early resection of the tumor.

## AUTHOR CONTRIBUTIONS

**Prachi Sharma:** Writing – original draft. **Yusuf A. Kumble:** Writing – original draft; writing – review and editing. **Abhigan Babu Shrestha:** Writing – original draft; writing – review and editing. **Vikash Jaiswal:** Writing – review and editing.

## FUNDING INFORMATION

None.

## CONFLICT OF INTEREST STATEMENT

None.

## CONSENT

Written informed consent was obtained from the patient to publish this report in accordance with the journal's patient consent policy.

## Data Availability

Not applicable.
